# Echo State Networks for Estimating Exteroceptive Conditions From Proprioceptive States in Quadruped Robots

**DOI:** 10.3389/fnbot.2021.655330

**Published:** 2021-08-23

**Authors:** Mario Calandra, Luca Patanè, Tao Sun, Paolo Arena, Poramate Manoonpong

**Affiliations:** ^1^Department of Electrical, Electronic and Computer Engineering, University of Catania, Catania, Italy; ^2^Department of Engineering, University of Messina, Messina, Italy; ^3^Institute of Bio-inspired Structure and Surface Engineering, College of Mechanical and Electrical Engineering, Nanjing University of Aeronautics and Astronautics, Nanjing, China; ^4^Embodied AI & Neurorobotics Lab, SDU Biorobotics, Mærsk Mc-Kinney Møller Institute, University of Southern Denmark, Odense, Denmark

**Keywords:** legged robot, echo state network, ground reaction forces, terrain classification, neural reuse

## Abstract

We propose a methodology based on reservoir computing for mapping local proprioceptive information acquired at the level of the leg joints of a simulated quadruped robot into exteroceptive and global information, including both the ground reaction forces at the level of the different legs and information about the type of terrain traversed by the robot. Both dynamic estimation and terrain classification can be achieved concurrently with the same reservoir computing structure, which serves as a soft sensor device. Simulation results are presented together with preliminary experiments on a real quadruped robot. They demonstrate the suitability of the proposed approach for various terrains and sensory system fault conditions. The strategy, which belongs to the class of data-driven models, is independent of the robotic mechanical design and can easily be generalized to different robotic structures.

## 1. Introduction

Legged robots complement wheeled machines because of the potential capability of the former to explore complex unstructured terrains. However, their effective use in practical environments has not become common because of several problems that are yet to be addressed. One primary issue is locomotion. Although several efficient control strategies have already been introduced in the literature (He et al., 2019), their main drawbacks are the lack of efficient high-performance sensing devices and processing techniques for obtaining the terrain characteristics in real-time. From this perspective, haptic feedback is a primary information source for achieving reliable locomotion in legged robots, especially in uneven terrains where real-time gait adaptation and attitude control are needed. The interaction with the terrain is commonly sensed through force sensors that estimate the ground reaction forces (GRFs) acting on the individual legs. Since the first reliable applications of locomotion control strategies in legged robots (Righetti and Ijspeert, 2008), multidimensional force sensors have been installed on robot feet to sense the ground reaction forces for closing the loop with neighboring ground locations. In Montes and Armada (2016), several strategies for force control were discussed. These strategies rely on signal acquisition from force sensors integrated within the mechanical structure of the robot feet without the use of expensive and bulky commercial sensors. In Bledt et al. (2018), a combination of impedance control and model predictive control was used to perform impressive tasks such as back-flips in a quadruped robot. These control methods require an accurate model of the terrain-leg interactions through contact force sensing or reliable estimation. In legged locomotion, ground reaction sensing at the individual foot level involves repetitive impacts with the terrain, which can easily affect and degrade the reliability of the device. Moreover, force signals detected by GRF sensors often suffer from multiple false detections, especially on uneven terrain.

For these reasons, researchers are increasingly studying reliable sensorless techniques to estimate the ground-foot contact information. In Karatsidis et al. (2016), a method to predict the GRFs in humanoid walking was presented. The method uses only kinematic information from a fully ambulatory inertial motion capture (IMC) system based on a large number of inertial motion units distributed over the body of the humanoid structure. Therefore, the enhanced accuracy of the force sensors comes at the expense of having many alternative sensor units. The force information can be obtained directly or indirectly from signals already available in the structure. The GRFs can be indirectly estimated from their inertial effects on the robotic structure, for example, from the torques or currents of the motors actuating the robot legs. In Bosworth et al. (2015), a classical approach involving the robot Jacobian matrix was used to estimate the foot force from the joint torques, which were in turn estimated from the leg actuator currents. Other approaches are based on Kalman filtering techniques or other methodologies derived from observations (Chan et al., 2013; Hu and Xiong, 2018). In Chenkun et al. (2015), a dynamic model of the leg structure was used for sensor estimation. The authors were aware that accurate parameter estimation is difficult to achieve. To match the actual robot results with the simulations, the unknown parametric uncertainties were identified through a learning process based on the actual data. In particular, radial basis function networks were used. Recently, a new method to estimate the force at the foot contacts was presented in Hu and Xiong (2018). The method is based on designing a generalized momentum observer for the robot force disturbances caused by the foot contacts on the ground. This method requires information on the joint positions and the applied control torques. The method is used to implement impedance control, in which the accuracy of the ground contact force is essential, especially soon after contact events, where the signals show large impulse-like variations. The methodology applied requires accurate knowledge of the system parameters, the most critical of which are concentrated on the robot structure and mass distribution. These parameters affect the estimation of the center of gravity motion. A deviation from the nominal parameter values can thus affect the overall performance of the method. For this reason, an additional neural network approach was used to compensate for errors due to inaccurate parametric modeling and dynamic effects. GRF estimation in legged machines has also attracted interest because of its potential applications in designing efficient prosthetic devices. In Fakoorian et al. (2016), the GRF was estimated on a leg prosthetic system using a Kalman filtering approach. Impedance control methods are often adopted to estimate the contact forces from trajectory tracking errors. In Xin et al. (2020), this strategy was applied as haptic feedback for teleoperation. The main hypothesis is to assume that the model error is much smaller than the disturbances.

All these approaches show that legged machines are complicated structures involving the concurrent motion of multiple bodies, each of which has its own inertial effects on the overall structure. Traditional approaches based on dynamic equations are thus not completely sufficient for accurate modeling. Moreover, in general, the classical approach is dependent on the particular robot structure used. Because of the extremely large variety of different legged machines described in the literature, tailored for specific tasks and applications, extracting accurate dynamic models for sensor estimation is a time-consuming and often complex task that is further complicated by the difficulty of accurately identifying the relevant parameters. Therefore, it is useful to employ a data-driven, neural network-based learning approach that accurately estimates the GRF sensor signals independently of the particular dynamic robot structure and acts as a reliable soft sensor device that can also cope with leg malfunctions. In Hwangbo et al. (2019), a strategy to train a neural network policy in simulation and then transferring it to a legged robot is presented. Specific attention was devoted to model the robot actuators. They are modeled through a data-driven approach, mapping the joint state and position error history into the torque signals provided to the simulated robot.

In our work, we explore the application of a family of recurrent networks to estimate the GRFs using proprioceptive local information acquired at the level of the leg joints. The underlining nonlinear dynamical model is defined after a learning process by extracting the temporal dependencies between the input data. The input data are projected into a pool of interconnected neurons called reservoirs in which both space and time-relevant information can be stored in an internal memory generated through recurrent connections. This methodology, usually referred to as reservoir computing (RC), represents an interesting approach for designing data-driven models in robotic applications involving nonlinear dynamic behaviors. Among the different architectures in the RC field, we selected the echo state network (ESN), which is commonly employed in various applications ranging from handwriting recognition (Bunke and Varga, 2007) to time series forecasting (Wang et al., 2019).

The concept of reservoir computing has been further extended in literature, from a pure algorithmic solution to include the physical device in the computational effort, realizing a physical reservoir computing system (Tanaka et al., 2019; Nakajima, 2020). An interesting demonstration was provided in Nakajima et al. (2015) where a soft silicone arm was adopted for real-time computation exploiting the intrinsic characteristics of the system including nonlinearity, memory, and potentially infinitely many degrees of freedom. Similarly, in Caluwaerts et al. (2014) a Reservoir Compliant Tensegrity Robot hardware prototype was presented; it was considered as a part of the computational system used to generate a set of desired oscillatory motor signals starting from a Matsuoka oscillator. The idea to use the robot dynamics to generate an embodied control system has been applied to a quadruped robot in Degrave et al. (2015). The main result of that work was to demonstrate that a memoryless feedback controller can generate a stable trot by learning the desired nonlinear relation between the input and the output signals.

A key advantage of ESNs compared with other neural structures is the simplicity of the learning process. The limitation of the learning process to only the output weights, called the readout map, significantly reduces the learning time. The increase in dimensionality due to the information transfer from the input to the hidden neurons, which present recurrent connections, produces multiple combinations of dynamics that can be exploited through the readout map depending on the task to be fulfilled. Moreover, the ESN approach is particularly advantageous over the other approaches mentioned above because it is a black-box identification model that avoids the need to implement model-based strategies that, in any case, would need to be refined with data-based learning algorithms. Our approach has additional advantages when dealing with joint faults. Adapting classical methods to handle such occurrences would be extremely complicated, whereas a learning-based technique based on recurrent ESNs allows the faults to be handled efficiently and provides information on the estimated GRF even when there is serious damage in the sensory system at the level of the joint legs. Similar capabilities were demonstrated in Antonelo et al. (2007) where a reservoir network was applied to a problem of robot localization and map creation, showing good performance also in presence of limited sensory information.

The use of ESNs also contributes to building an internal memory that is particularly useful for handling time-varying signals. An additional important issue typical in reservoir computing networks results from the characteristics of the dynamics processing in the reservoir layer. The latter utilizes a sparse representation of the input signals in a high-dimension dynamical projection space, whereas the readout maps constitute only a low-dimension projection space, defined after the learning phase, that maps specific aspects of the input features. In principle, any set of information consistent with a given input signal can be extracted from a given reservoir lattice in parallel through the addition of other readout maps. This is a typical example of *neural reuse* (Anderson, 2010; Arena et al., 2013). In this work, a clear application of these characteristics is presented, and another readout neuron is added to the same neural lattice used for GRF estimation to classify the type of terrain traversed by the robot (i.e., flat, downhill, uphill).

There are different approaches in the literature related to the design of solutions for terrain classification in legged robots in particular, in relation to the material type. In Hoffmann et al. (2012), a sensory-motor classification of different terrains was presented for a quadruped robot. The role of the action context to further improves the discrimination capabilities was also demonstrated. Techniques based on extreme learning machines and reservoir computing were analyzed in Degrave et al. (2013) to demonstrate the effectiveness of a limited combination of tactile and proprioceptive joint sensors for terrain classification. These studies can be framed within the embodied cognition framework: the idea is to find the emergence of proto-cognitive behaviors letting the robot extracting regularities in the sensory-motor space and exploit them for action generation (Hoffmann, 2014). To analyse the flow of information in sensorimotor networks, tools from information theory were adopted in Schmidt et al. (2013). The results demonstrate the possibility to create a primitive body schema identifying structures in the sensorimotor space.

In our work we are presenting a unique network able to provide both the GRF distribution on the legs and the terrain slope with high classification accuracy. This information could be used, for example, to select the most appropriate locomotion gait for the application. Preliminary experimental results, carried out on a real quadruped robot, demonstrate the effectiveness of the proposed approach. Furthermore, although the embedded hardware implementation is not within the scope of this work, the authors identified potential solutions to develop embedded ESN structures.

A first attempt is reported in Huang et al. (2019) where a scalable RC-ESNs hardware generator for embedded computing is presented. The strategy consists of a high-level synthesis in conjunction with design automation to automatically transform an offline-trained ESN algorithm into an embedded hardware accelerator for FPGA applications. Problems related to efficiency in terms of power, performance, and occupied area were also considered and addressed. This approach is in line with another recent example that follows this hardware-oriented strategy (Huang et al., 2020): here an automatic holistic energy-aware design methodology is proposed and applied to a multilayer perceptron designed to be embedded in proactive brain-machine interface edge devices based on FPGA. Another interesting direction for hardware implementation is related to the open-source Neural Network framework called Neural Network on Microcontroller (NNoM)^1^, for implementing (recurrent) neural networks on a microcontroller. It provides a user-friendly interface and supports state-of-the-art neural model structures. However, the chip market is rapidly changing and new opportunities (e.g., System-on-a-chip, tensor computers, and neuromorphic hardware) will be more and more available in the next years.

The remainder of this paper is organized as follows: The methodology employed in the paper is introduced in section 2, in which the robotic structure and the ESN structure used for the sensor signal estimation are also presented. Simulation results for both the GRF estimation and terrain classification are reported in section 3. The application of the ESN for GRF estimation to a real robot is discussed in section 4. The work is concluded and some perspectives are provided in section 5.

## 2. Methodology

The aim of this study is to employ reservoir computing structures to predict external signals, such as the leg ground reaction forces, using internal data such as the joint torques in a quadrupedal robot structure. All the data to be analyzed were acquired on a simulated robot moving in a dynamic simulation framework named CoppeliaSim, which has been duly extended in Rohmer et al. (2013). The framework provides an accurate dynamic simulation environment that is particularly useful for complex robotic structures. The simulation approach becomes essential when sophisticated learning-based control techniques, which involve time-consuming runs, have to be applied to the structure before obtaining reliable results. To achieve the aim of the study, the training phase of the methodology introduced here was first performed in the dynamic simulation before implementation on the actual robot prototype. The simplified foot structures in several-legged robots do not allow the inclusion of GRF sensors, which are useful for developing adaptive locomotion control strategies. One example is the Lilibot robot, which is a small-sized robot developed for research and education purposes (Sun et al., 2020). The first attempt to solve the GRF acquisition problem adopted a simple parametric model utilizing the current through the servo motors at the knee joints as the input signal; the current was found to be positively correlated with the GRF. Our work extends this approach, which is based on a static model, by developing a dynamic structure that can utilize the time evolution of signals relevant to the joints, in particular the torque signal of a subset of joints, to estimate the GRF. The linear relationship between the joint torques and motor currents ensures that the proposed model can be applied in the robot to easily acquire information on the currents absorbed by each motor. Moreover, a significant improvement over the model in Sun et al. (2020) is the development of a unique network that utilizes the information coming from all four legs to estimate the GRF signals. This approach allows local faults within the joint sensory acquisition system to be handled and provides a good reconstruction of the GRF associated with a leg even if the corresponding joint signals are not available. The additional update is made possible by using the same reservoir lattice to provide information about the type of terrain the robot is actually walking on.

### 2.1. Lilibot Robot

**Figure 1:**
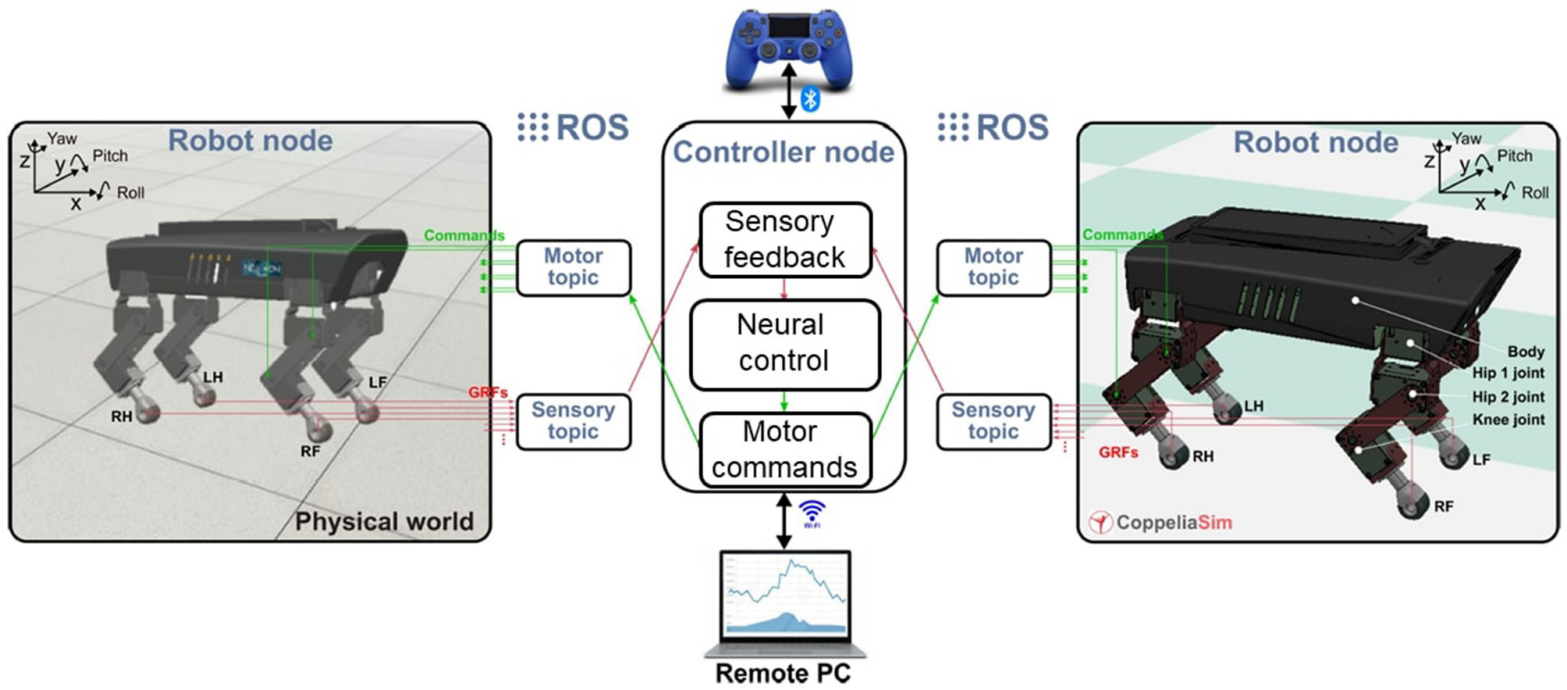
Overview of the 12 DoF quadruped Lilibot. Lilibot has a software framework with a modular design. The framework is based on the robot operating system (ROS) and can be connected to a joystick and a remote computer for manual control and robot state monitoring/recording. The simulated and physical versions of Lilibot are identical. A control mechanism can be first tested on the simulated robot and then directly transferred to the actual robot. Further details are reported in Sun et al. (2020).

Lilibot is a small, lightweight, robust, open-source, and sensor-rich quadruped robot (Sun et al., 2020) (Figure 1). Each leg is characterized by three joints comprising two hips and a knee, as shown in Figure 1. The flexible configuration of the robot leg allows extensive rotation at the level of the joints and results in large workspaces due to the small dimensions of the robot. This makes the structure an ideal platform for studying adaptive locomotion strategies. An algorithm capable of estimating the GRF for each leg of the actual robot through the knee currents was also provided in the paper referenced above. In the present study, torques were used instead of currents for GRF estimation because of the lack of information on the actuator currents in the robot simulator. To demonstrate the reliability of the results obtained, different simulations were performed with varying characteristics of the ground the simulated robot walked on. Data were acquired not only for a flat surface but also for an uphill surface and a downhill surface. We focused on measuring the joint torques and the leg GRFs.

The robot operating system (ROS) was used to create a communication channel between the controller and the simulated robot. The locomotion controller is an adaptive neural controller written separately from the simulation environment. It communicates with the simulated robot through specific channels called topics which are provided by the ROS. CoppeliaSim allows some robot parameters such as the leg joint torques and the leg GRFs to be monitored. The simulation was constructed such that it almost perfectly reflected the behavior of a real robot (Sun et al., 2020). The adopted locomotion control system is a central pattern generator (CPG) which can be adapted to generate different locomotion gaits through a series of parameters. In the following simulations, the robot walked at a fixed speed with a trot gait in which two opposite legs were in phase at each moment while the other two legs were 180° out of phase.

The CPG was devoted to low-level locomotion control, whereas the high-level ESN structure was implemented for GRF estimation and terrain classification. Therefore, the next step consisted of configuring the ESN where a different set of parameters was used to find the most reliable model that provided the best results. Different architectures were used based on the number of legs and type of joints tested during the analysis. In the following step, the robustness against faults was analyzed to train a model that can reliably react to sudden faults affecting the leg joint sensory system.

### 2.2. Echo State Network Overview

**Figure 2:**
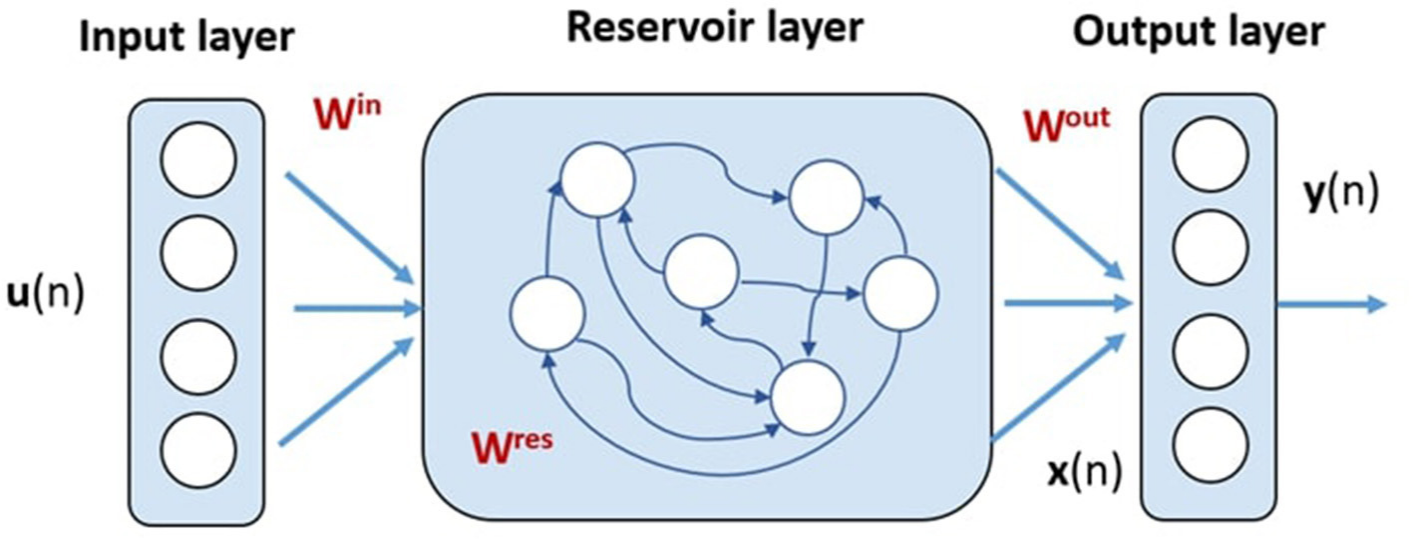
Echo state network structure comprising an input layer, a reservoir layer, and an output layer. Only the *W*^*res*^ weights are subject to learning.

An echo state network proposed in the early 2000s (Jaeger, 2001) was used to predict the GRF of each leg joint torque reading. This specific neural architecture falls within the field of reservoir computing, which is a collection of methodologies useful for training recurrent neural networks. A reservoir computing system consists of a reservoir that maps input signals into a high-dimensional space and a readout map for pattern matching from the high-dimensional states in the reservoir to an output target. A simple scheme for this architecture is presented in Figure 2.

The advantage of reservoir computing ESNs is that, whereas the reservoir layer (which corresponds to the hidden layer in feedforward networks) has random fixed weights, only the readout is trained with simple methods consisting of, for example, the recursive least square (RLS) algorithm. Thus, the major advantage of reservoir computing compared to other recurrent neural networks is fast learning, which results in low training costs (Tanaka et al., 2019; Patanè and Xibilia, 2021). This study aims to show how powerful and lightweight an ESN can be in the development of a soft sensor for robotic applications. The reservoir can be conceived as a bucket of neurons, each of which is sparsely connected to other internal neurons. The output neurons are all connected to individual reservoir neurons, whereas the input neurons are sparsely connected to the reservoir neurons. Each connection is described by a uniformly sampled random weight value. However, during the training phase, only the readout weights are trained to improve the model accuracy (Lukoševičius and Jaeger, 2009). This is the main characteristic that allows the ESN to be lightweight. In the absence of feedback from the output to the reservoir, the time evolution of the neuronal states in the reservoir is given by Jaeger (2001).

(1) $$\begin{array}{l} x\left(n\right)=\lambda f\left(W^{in}u\left(n\right)+W^{res}x\left(n-1\right)\right)+\left(1-\lambda \right)x\left(n-1\right) \end{array}$$

where *n* denotes the discrete time, ***x***(*n*) the state vector of the reservoir units, ***u***(*n*) the input vector, *W*^*in*^ the weight matrix for the input-reservoir connections, and *W*^*res*^ the weight matrix for the recurrent connections in the reservoir. Function *f* represents the element-wise activation function of the reservoir units and λ ∈[0, 1] is the leak term, adopted when leaky integrator neurons are considered. In our case study, we chose the hyperbolic tangent as the activation function. The output is given by a linear combination of neuronal states:

(2) $$\begin{array}{l} y\left(n\right)=W^{out}x\left(n\right) \end{array}$$

where ***y***(*n*) is the output vector, and *W*^*out*^ is the weight matrix in the readout. In supervised learning, this weight matrix is trained to minimize the difference between the network output and the desired output for a certain time period (Lukoševičius and Jaeger, 2009).

An ESN is characterized by a set of parameters that are directly connected to its behavior. We tested different parameters to determine the model with the best accuracy. We provide the values of the key network parameters in Table 1 that summarizes the relevant characteristics of the proposed architecture and the hyperparameters adopted. The selection of these hyperparameters was driven by the indications available in literature (Bengio, 2012; Dasgupta, 2015; Dasgupta et al., 2015) and by preliminary experiments. Therefore, it was performed through a trial-and-error procedure based on a combination of expert knowledge to identify a searching domain, and a grid search performed on the reduced subspace of the hyperparameters to identify the best configuration in terms of prediction accuracy on the validation dataset. As a result, the reservoir neurons were set to 100 based on our previous analysis (Dasgupta et al., 2015) and grid search. The leak parameter, defining how much a single neuron in the network depends on the actual net input it receives, was analyzed in Dasgupta (2015) and Dasgupta et al. (2013) and here set to 0.3 based on the analysis. Note that a smaller value will lead to less leak of the information, i.e., larger temporal memory storage while a larger value will lead to high leak of the information, i.e., smaller temporal memory storage. The input sparsity defines the probability of connections from the input to the network which was empirically set to 20%. This provides robustness to the network and less input dependent compared to a higher sparsity value (Dasgupta, 2015). The network sparsity defines the connection probability between reservoir neurons. It is typically set to 10–50% (Dasgupta, 2015). Here it was empirically set to 50%. The spectral radius parameter (or network scaling factor) was analyzed in Dasgupta et al. (2015) and Dasgupta et al. (2013). Based on the spectral radius analysis, the parameter was set here to 0.95 such that the spontaneous network dynamics is in a stable regime and achieves the best performance of the chosen network size. The constant noise bias (i.e., 0.001) is applied to the hidden recurrent neurons of the network. The bias term is set based on Rungruangsak-Torrissen and Manoonpong (2019) and used in order to provide a small input for the hidden neurons to constantly activate them, thereby maintaining the neurodynamics. The other parameters, like learning rate and washout, were set with respect to the standard setup of the ESN learning (Bengio, 2012; Dasgupta et al., 2013, 2015).

**Table 1:** Echo State Network parameters which provided the best accuracy.

**Parameter**	**Value**
Reservoir neurons	100
Learning method	RLS
Leak	0.3
Learning rate	1.0
Input sparsity	20%
Network sparsity	50%
Spectral radius	0.95
Reservoir function	Tanh
Readout function	Linear
Washout	100
Noise bias	0.001

Other hyperparameter optimization methods, based on genetic algorithms and different bio-inspired approaches, have been applied in recent works and can be considered as further searching strategies (Tian, 2020).

A particular point of interest is the choice of the learning method (here, the RLS) and the spectral radius. The spectral radius is related to the Echo State Property, an important property that guarantees the stability of the network that is able to forget its inputs after a given time behaving as a fading memory. The spectral radius is usually kept below 1 to maintain the echo properties for zero input reservoirs. This constraint is usually enough for a large reservoir (Caluwaerts et al., 2013), although, in some application, the possibility to explore the range above 1 could be useful to improve the network generation capability of chaotic signals (Sussillo and Abbott, 2009). In presence of input-driven reservoirs, temporal and statistical properties of the driving input can be related to the spectral radius that may exceed the previous mentioned limit by continuing to hold the echo state property (Manjunath and Jaeger, 2013). As stated above, the training of an ESN is relatively faster than that of standard recurrent neural networks (Hochreiter and Schmidhuber, 1997; Mandic and Chamber, 2002).

We considered a standard ESN architecture to demonstrate the effectiveness of our strategy. However, further investigations to improve the proposed model performance could consider the introduction of the intrinsic plasticity rule to adapt the reservoir internal parameters using an unsupervised mechanism based on the maximization of the transferred information (Dasgupta et al., 2013, 2015; Dasgupta, 2015; Patanè and Xibilia, 2021).

## 3. Simulations and Experimental Results

**Figure 3:**
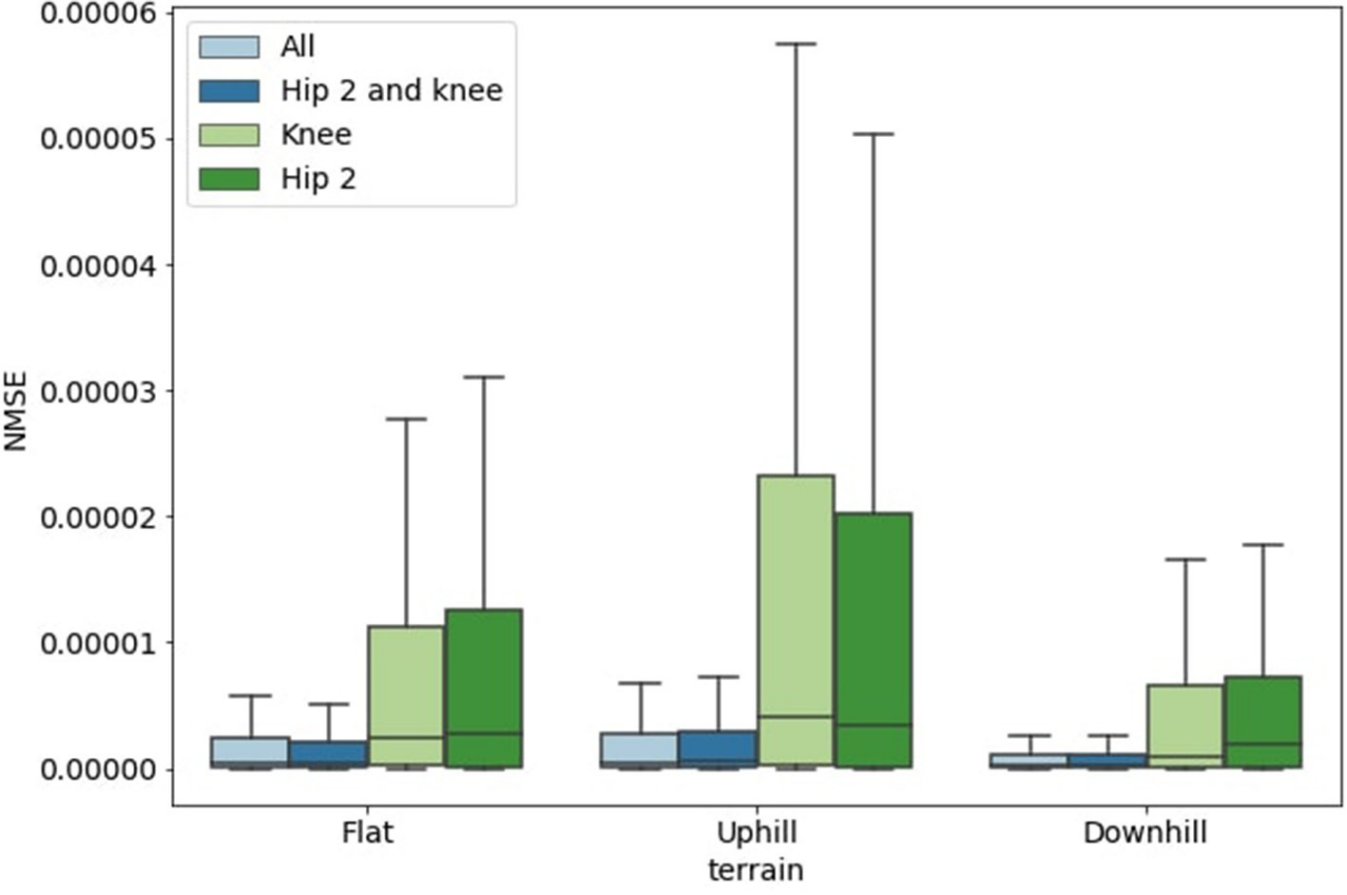
NMSE obtained from the ESN for different terrain types. The role of the input signals was investigated considering four different cases: all (three torque signals for each leg for a total of 12 inputs), Hip 2 and Knee (8 inputs), Knee (4 inputs), and Hip 2 (4 inputs).

The first step consists of data acquisition. The simulation was run for several minutes on perfectly flat ground. The robot walked at a fixed speed and gait. Both joint torques and the GRF of each leg were recorded with a sampling interval of 50 ms. The same operations were performed on both downhill and uphill ground with slopes of ±5° to verify the ability of the network to generalize the GRF prediction independently of the ground shape. After collecting all the data, a pre-processing stage was implemented. The final step was to train and test the model, followed by data analysis, which led to the results reported below. Because the focus was to obtain a good estimation of the leg GRF, we analyzed what proprioceptive information should be used to achieve the most reliable results. The analysis was performed systematically by using different sets of joint torques in the input layer. Each round of analysis was performed by first training the model on 80% of samples measured on flat ground and then testing the model on the remaining 20% of the flat ground dataset together with the complete uphill and downhill datasets, which were not shown to the model during the learning phase. The size of the entire dataset was ~25, 000 samples when the sampling rate was 20 Hz. The variables were normalized into the range [0, 1]. We measured the mean squared error (MSE) and its normalized version (NMSE), according to the set of joints provided to the model and the different tested surfaces. Figure 3 depicts the ESN performance in the two cases when all 12 joint torque signals were provided in the input, and when the input layer was reduced to only 8 signals (i.e., only the Hip 2 and Knee joints). The statistical results obtained indicate that the information from the Hip 1 joint is not relevant to the analysis. This result can be explained by the leg kinematics shown in Figure 1. Here, the Hip 1 joint is required only for steering or attitude controlling maneuvers, whereas the other two joints are involved in generating the stance and the swing trajectory on the sagittal plane during forward walking. A further reduction of the input signals to only one single-joint signal for each leg produces a drastic increase in the reconstruction error. This indicates that the optimal network configuration should include a total of 8 inputs consisting of the torque signals for the Knee and Hip 2 joints of all the legs. The outcomes of the test phase are very similar for all the terrain configurations, even when the model was trained using only data acquired on the flat surface. Thus, our model can be generalized to generate predictions for different terrain characteristics.

Another relevant characteristic of the proposed architecture that we investigated is its robustness against random faults in the torque sensors. The idea is to verify whether the model can handle the partial omission of some input signals and show a gradual performance degradation instead of an abrupt drop. The acceptability of the predicted quantities depends on the predicted error.

**Figure 4:**
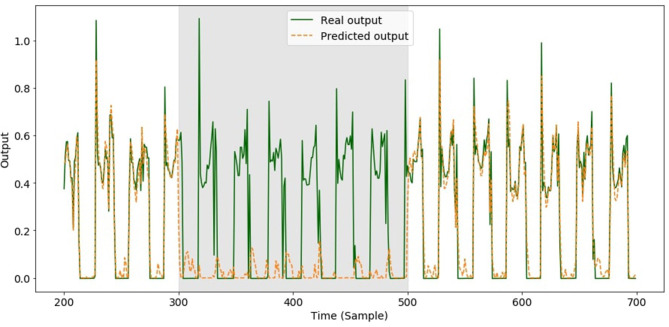
Predicted GRFs of front right leg when the model learnt to predict all GRFs and a fault was introduced over 200 samples (gray area from 300 to 500) in both of the front right leg joints. The training set consisted of data measured using perfectly working sensors.

We first introduced faults during the test phase. The faults affected both the Knee and the Hip 2 sensors in the front right leg for 200 consecutive samples, which corresponds to ~6 steps. A comparison between the actual and predicted GFR for the front right leg subjected to the fault is shown in Figure 4.

As can be seen, the test performance in the presence of the sensor faults is very poor; therefore, our next step consisted of finding a solution to avoid or at least limit the performance degradation. We thus evaluated the behavior of our final model trained in the presence of faults to find a good strategy to improve the accuracy of the predictions. One of the functions of the reservoir layer is to create hidden time correlations between the input joint signals, which can then be exploited by forcing the net to estimate the GRF of a leg even in the absence of torque signals from that leg. This can be achieved if the network learns the correlation between the corresponding leg joints and their involvement in the output prediction. The presence of a correlation between the joint torques of a leg and the GRFs of the other legs is reasonable because the robot is moving with a fixed gait. Therefore, we introduced artificial faults in each leg during the training phase. Each fault lasted for 100 samples and occurred in the Knee and the Hip 2 signals of each leg once every 500 samples. Situations involving faults in two or more legs at the same time were not considered.

Table 2 summarizes a statistical analysis of the prediction performance of the ESN-based model when the training and test phases were carried out with and without faults. The network can be forced to create cross relations between sensory information by introducing faults during training to significantly improve its fault-handling performance through the support of the available sensory signals coming from the other legs. This effect is obtained at the cost of a slight degradation of the prediction performance in the absence of faults.

**Table 2:** Mean and standard deviation of MSE in testing evaluated on all legs when the training and testing phase is performed in presence of faults as discussed in the text.

**Trained with fault**	**Tested with fault**	**MSE**	**R**
No	No	0.0036 ± 0.0015	0.98
No	Yes	0.0175 ± 0.0229	0.67
Yes	No	0.0056 ± 0.0004	0.96
Yes	Yes	0.0074 ± 0.0031	0.93

*The Pearson correlation coefficient (R) is also reported*.

**Figure 5:**
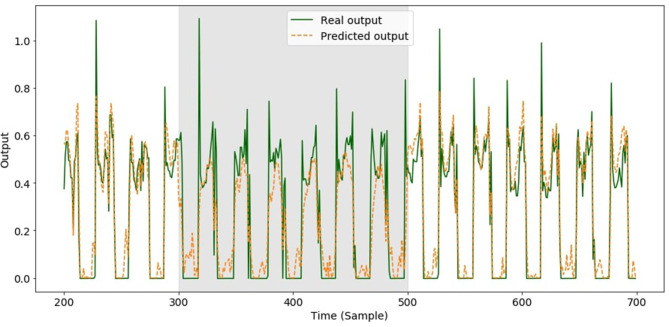
Predicted GRFs of front right leg when a fault was introduced in both front right leg joints over 200 samples (gray area from 300 to 500). The training set consisted of data that included the faults.

Figure 5 shows the improvement obtained for the GRF prediction of the faulty leg with the new training compared with the results previously reported in Figure 4.

**Figure 6:**
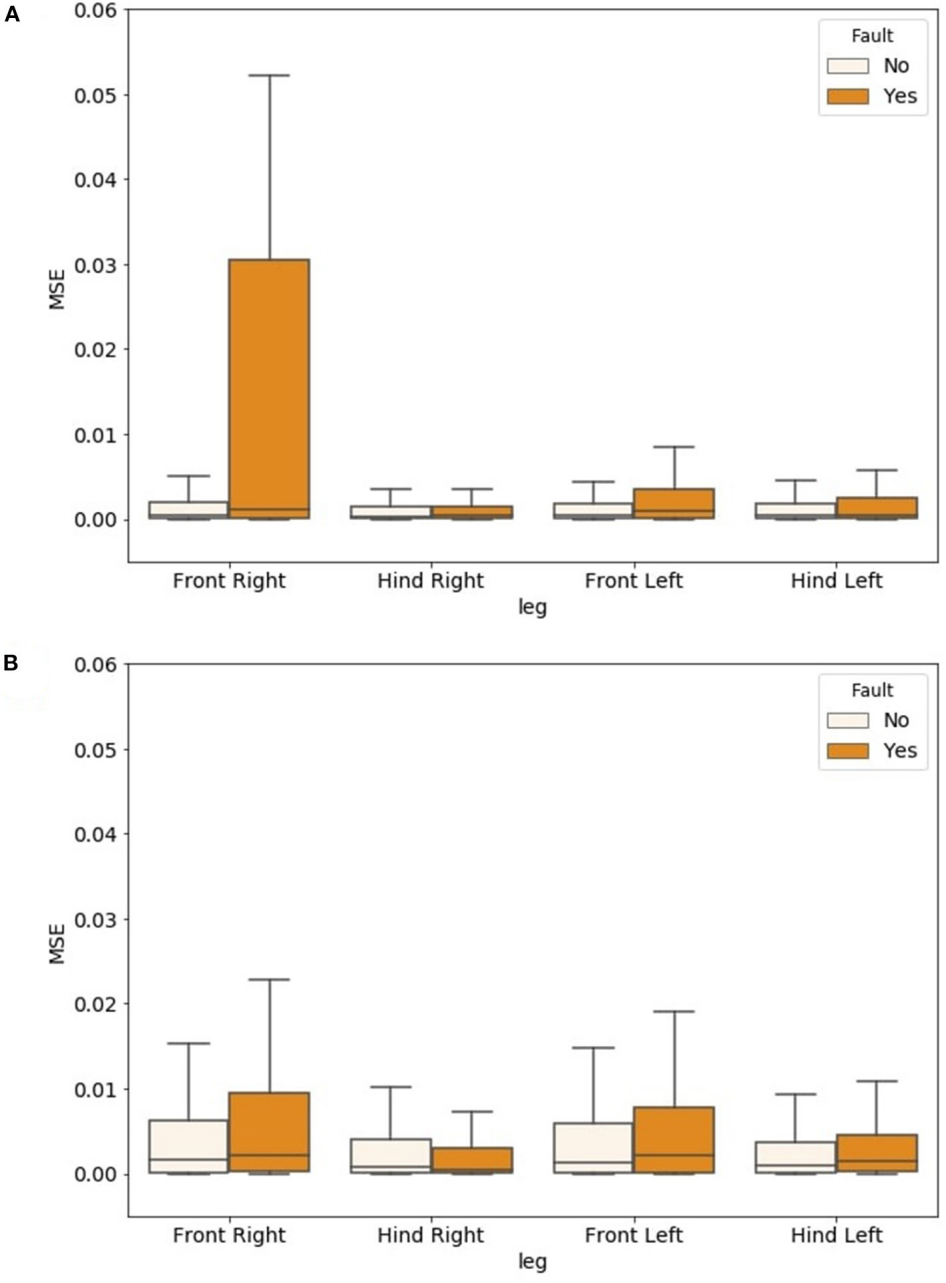
Comparison of prediction errors between samples with and without faults for the four GRF signals predicted as output of the ESN. The model was trained using only data from normal working conditions **(A)**, and using data in which artificial faults were introduced in all the legs during the training phase **(B)**.

These results can be analyzed in detail by considering the prediction error obtained for each output variable (i.e., each leg). Figure 6A shows the effect of a fault on both joints of the front right leg compared with the MSE obtained when all the sensory information was provided in the input. The degradation is evident and concentrated on the corresponding leg. The effect of the same fault on the model trained with faulty signals is shown in Figure 6B. Here, all the legs cooperated in predicting the four outputs, improving the robustness in the presence of faults.

**Figure 7:**
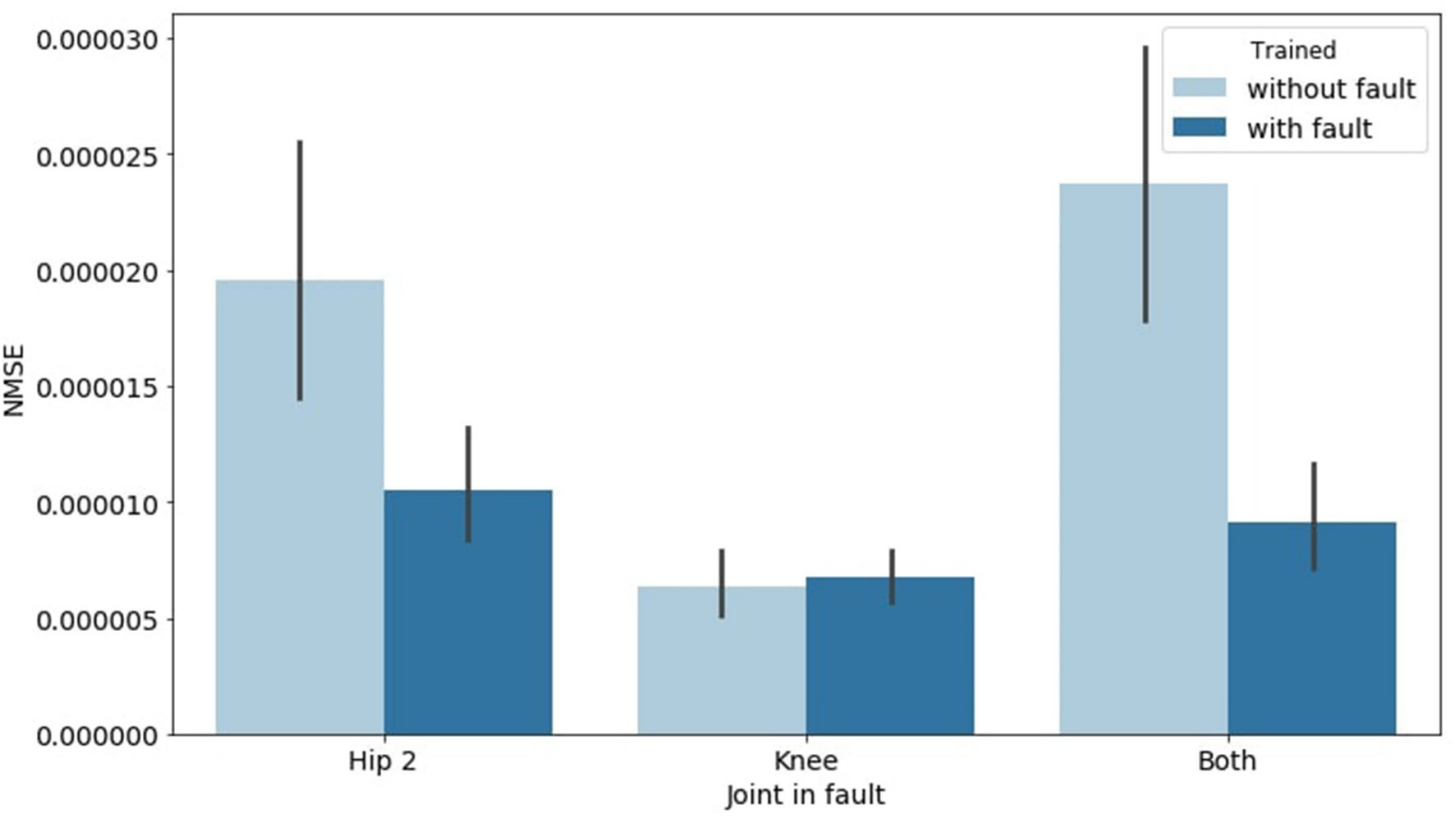
Behavior of GRF prediction NMSE when the fault occurred on Hip 2, Knee, or both front right leg joints. The error was computed as the average of all four legs. The brighter bar shows the measured error when the model was trained without artificial faults, whereas the darker one was obtained from a model trained with artificial faults.

**Figure 8:**
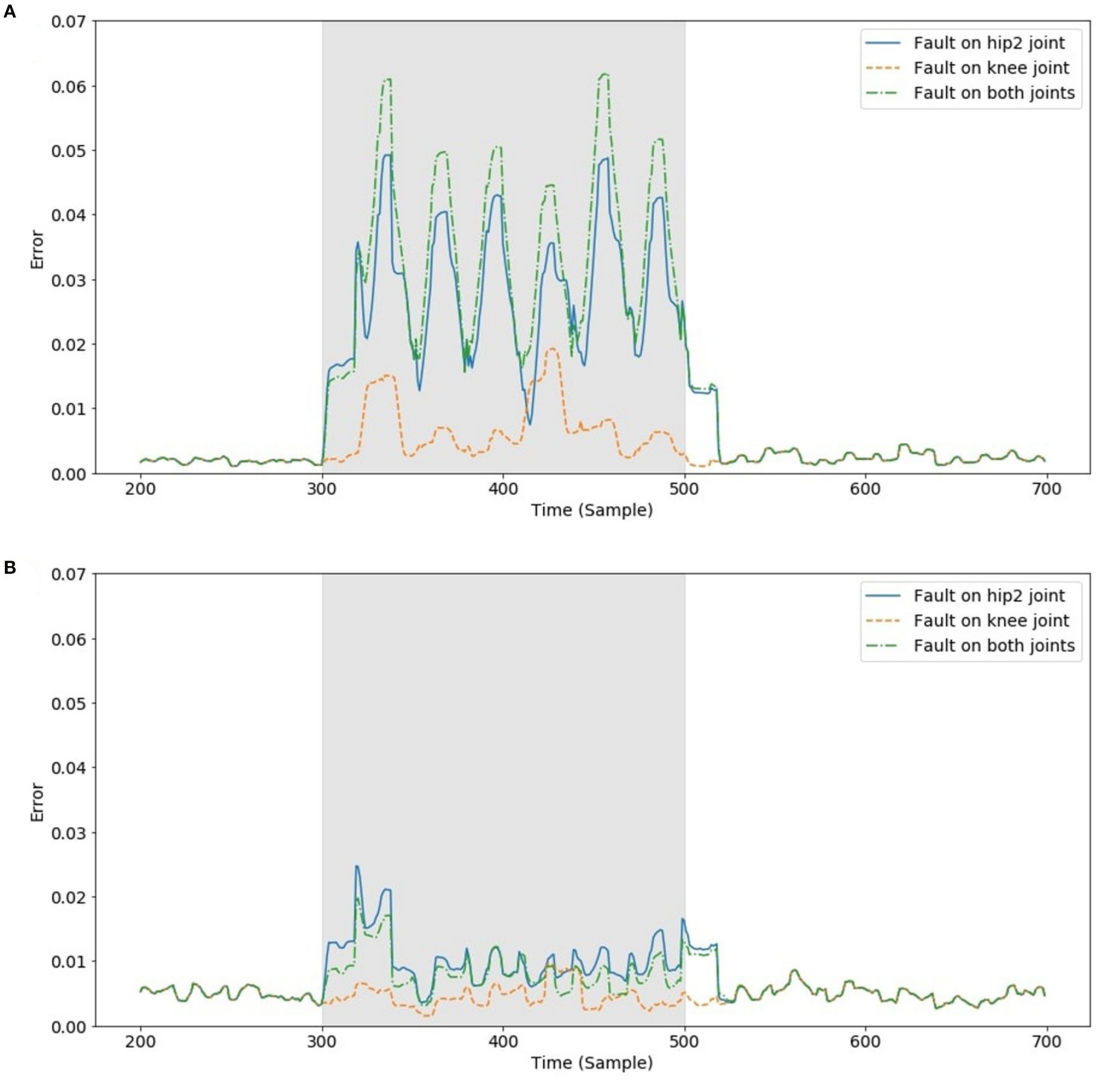
Absolute error between actual and predicted GRF of the front right leg over time when a fault occurred on a specific joint (gray area) and the learning was performed without **(A)** and with faults **(B)**. The error signal was filtered using a sliding window of 20 samples to facilitate comparison.

Similar conclusions can be drawn when a fault occurs on only one joint of a leg, given that the model was trained against faults occurring in the Hip 2 and Knee of each leg. Figure 7 shows how the NMSE behaves differently when the faults occur on Hip 2, Knee, and on both front right leg joints during the test over 200 consecutive samples. The error was computed as the average of all four legs. The brighter bar shows the measured error when the model was trained without artificial faults, whereas the darker one was obtained from a model trained with artificial faults. It is clear that the mean error is generally much lower if the model has learnt how to deal with faults. Figure 8 also shows how the error behaves over time when the faults occur on a specific joint. This result, combined with the previously analyzed results, suggests that there is a weak relationship between the leg knee torque and its GRF.

### 3.1. Terrain Classification

**Figure 9:**
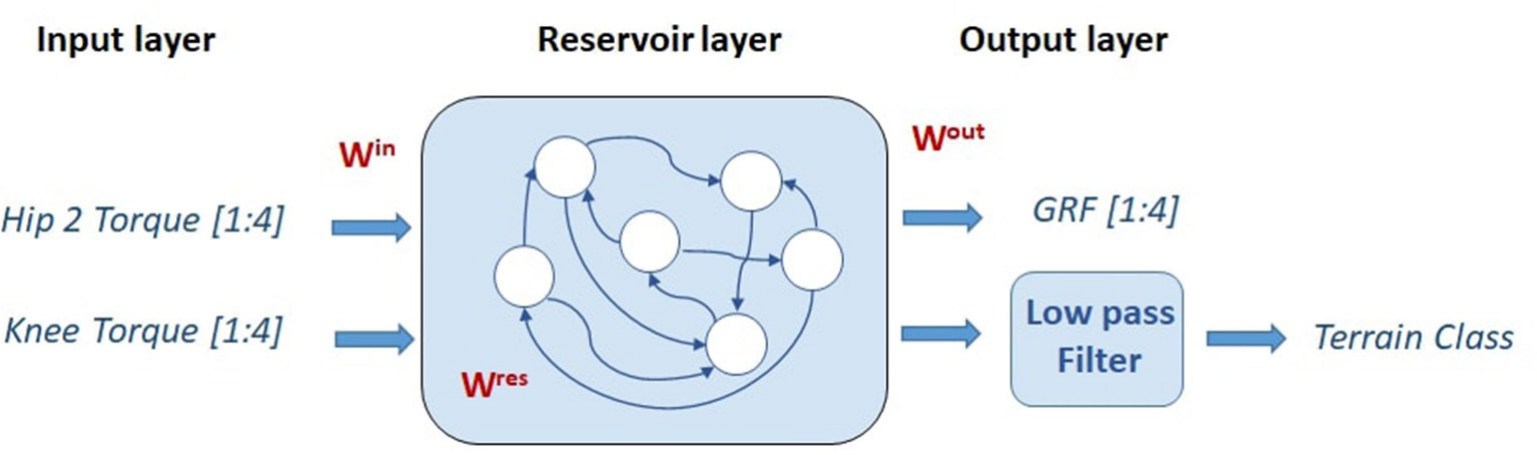
Echo state network structure extended to include the terrain classification task. The input layer includes eight torque signals coming from the Hip 2 and the Knee joints for all the legs, and the output layer contains two readout maps which are respectively dedicated to estimating the GRF in the four legs and classifying the terrain type into three categories (flat ground, uphill, and downhill). In this last case, a low-pass filter was adopted.

The results reported in this section show one of the most intriguing features of ESNs. As described above, the dynamics that emerge within the reservoir lattice do not depend on the target to be mapped; rather, they spontaneously arise as a result of the input signals and the random sparse arrangements of the connections and weights. Once these factors are fixed, the high-dimensional dynamics within the neuron lattice can be mapped according to an arbitrary assignment imposed by the target signals. This feature can be exploited to allow the use of a given dynamical arrangement to create many arbitrary mappings of the same input space for the realization of other readout maps. The example reported here uses the same dynamical input signals to obtain, in addition to the time-dependent GRF signals, a classification of the type of terrain traversed by the robot (i.e., flat, uphill, or downhill). This application exploits the relationship between the average slope of the climbed terrain and the interplay between the complex inertial effects caused by multibody motion and the motor torque distribution among the robot legs. Once the former ESN is trained, it is no longer necessary to run the entire network again. It is sufficient to add another readout map and exploit the output of the reservoir lattice to perform the desired mapping by training only the added map. Therefore, the terrain classification step was performed using the same network configuration and parameters used in the previous task. The new readout is extremely simple: it comprises only one output neuron that provides, as output, the three terrain types considered. This approach falls within the psychological paradigm of neural reuse (Anderson, 2010) recently adopted for neuro-inspired structures (Arena et al., 2013): neurons, because of their interconnectivity, organize in networks that can cope with different tasks concurrently. In our case, the same ESN network can generate multiple parallel signals from a single set of input signals previously adopted for robotic applications ranging from time-dependent GRF estimation signals to static labels that account for the type of terrain currently being traversed Figure 9 depicts the augmented ESN structure, which includes the terrain classifier. The reservoir layer is the same layer as that for the GRF estimation, and only the additional readout map is trained.

The target for each class is a constant value: 0 for downhill terrain, 0.5 for flat terrain, and 1 for uphill terrain. In the last case, a low-pass filter was adopted at the output stage to provide a smooth signal. In particular, the output of the ESN was processed using a 5th-order Butterworth filter with a cutoff frequency of 0.5 Hz. The introduction of nonlinearities in the output layer will be investigated in future works to avoid the presence of an external filter.

The classification was performed by considering the average output over a time window of 100 samples. The average error between the network output and the three target signals was computed, and the class with the smallest error was selected for the current window. As stated above, the ESN with the same topology as that in Table 1 was considered. In addition, different datasets were considered in evaluating the capability of the network to classify the terrain when there was missing information in the input signals. When all the input information is available the classification accuracy is 100%. However, the classification solution cannot easily handle sensory fault events. In fact, if the torque signals of one leg are missing, the performance decreases, and the accuracy is reduced to 76.6%. Similar to the GRF estimation discussed above, to improve the network prediction performance in the presence of faults, the learning dataset was modified to include faults. The resulting accuracy of the network when faults were present on both the Hip 2 and Knee joints of a single leg reached the high value of 97.6%.

**Figure 10:**
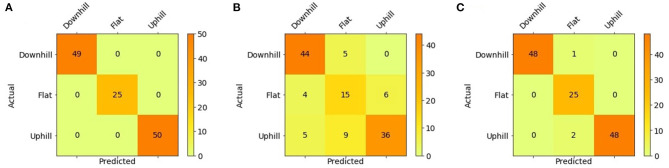
Confusion matrices obtained for different data configurations: **(A)** learning without faults and testing without faults, **(B)** learning without faults and testing with faults, and **(C)** learning with faults and testing with faults. The configuration learning with faults and testing without faults was characterized by a perfect classification and is equivalent to the confusion matrix in **(A)**.

**Figure 11:**
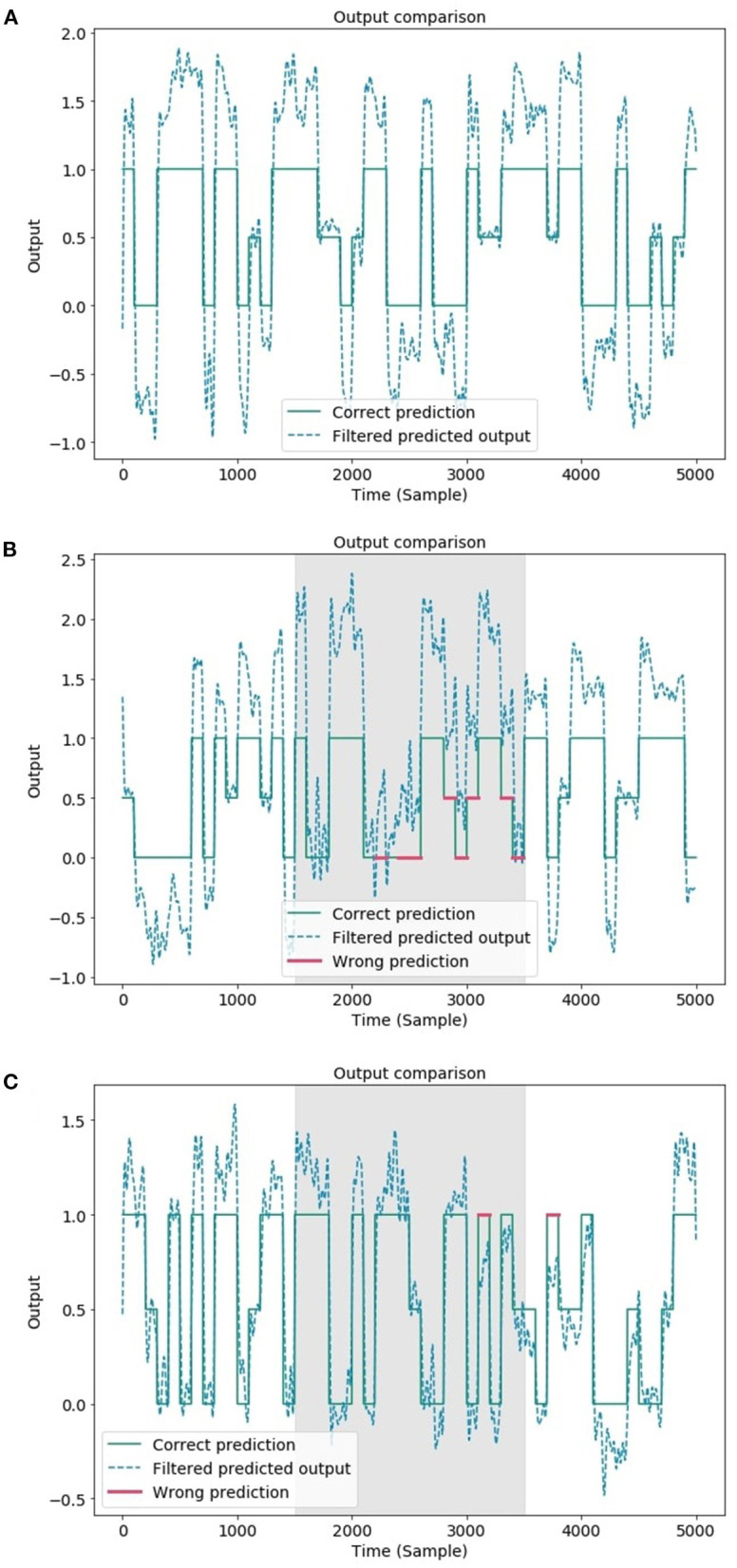
Comparison between the filtered predicted output and the target signal obtained for different data configurations: learning without faults and testing without faults **(A)**, learning without faults and testing with faults **(B)**, and learning with faults and testing with faults **(C)**. The fault consists of the unavailability of the sensory signals coming from the front right leg (Hip2 and Knee joints) for 2,000 samples (gray area).

The confusion matrices obtained during the testing phase for the three different cases considered here are reported in Figure 10. A total of 124 time windows were analyzed. The filtered output of the network is compared with the actual class in Figure 11. The classification accuracy is considerably reduced when the presence of faults results in multiple incorrect predictions. The addition of faulty conditions in the training dataset for the learning procedure improves the network performance and drastically reduces the number of incorrect predictions.

### 3.2. Real Robot Experiments

**Figure 12:**
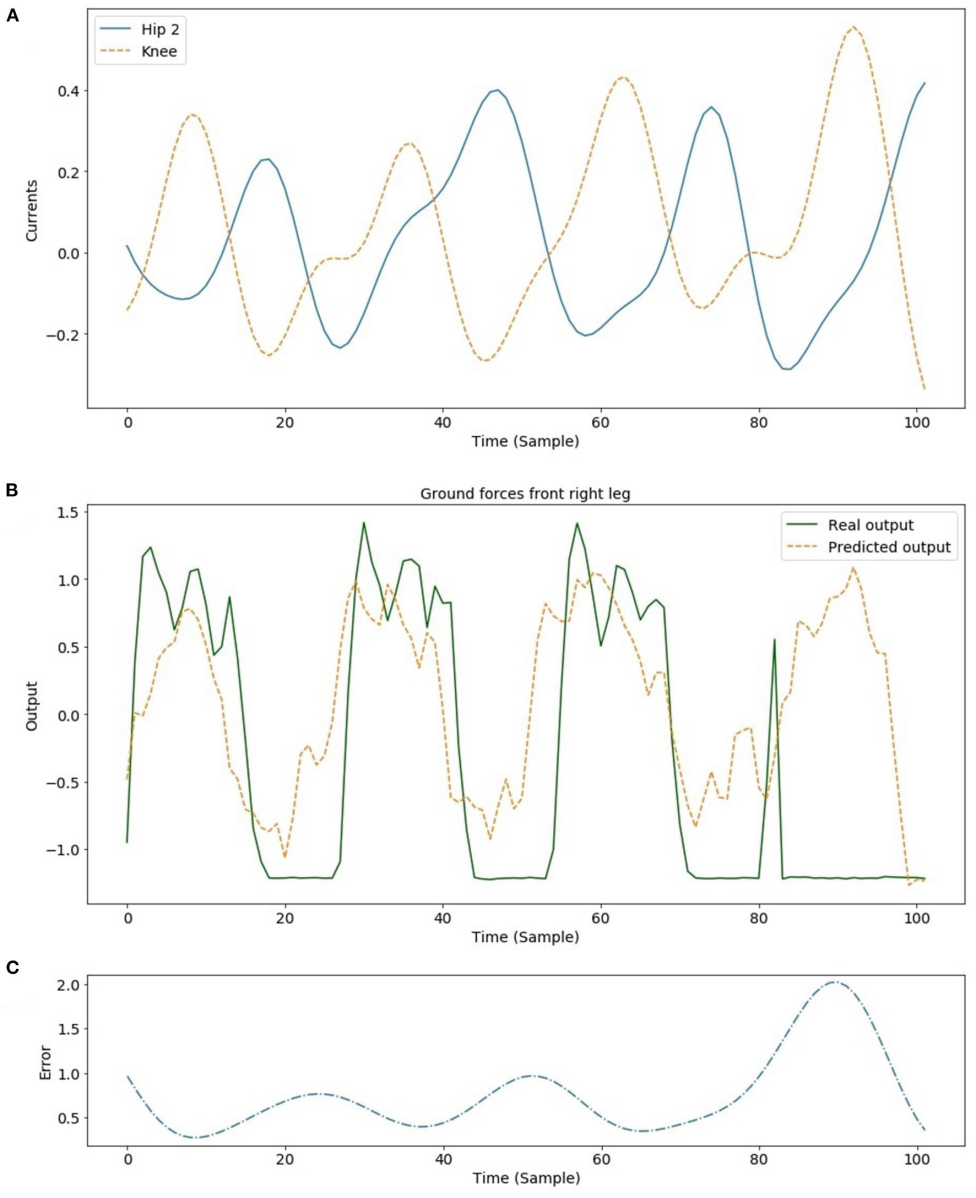
Input and output signals for the ESN trained to predict the GRF of the front right leg of the Lilibot quadruped robot: **(A)** the trend of the filtered motor currents on the Hip 2 and Knee robot joints; **(B)** the comparison between the estimated and actual GRF acquired from the real robot during the testing phase; **(C)** the prediction error where the presence of an unexpected situation is highlighted by the increased values in the error signal.

**Figure 13:**
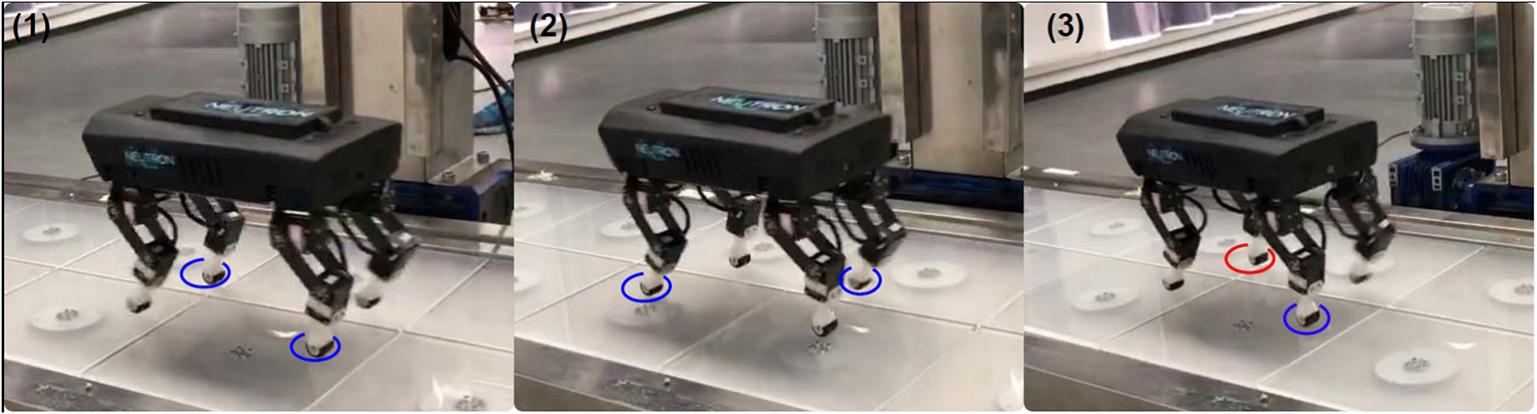
Snapshot of the Lilibot walking on the force plate. The blue circles indicate the stance feet touching on a single measurement unit while the red circle indicate the feet (i.e., right front foot) touching on the boundary of two measurement units in which the force plate could not identify the GRF acted on the foot (i.e., right front foot).

The proposed approach for GRF estimation was particularly effective on the simulated quadruped robot. To further assess the ESN-based strategy, an experimental set-up was considered to properly acquire the needed data from the Lilibot robot, as the current robot setup does not include GRF sensors (Sun et al., 2020). The experimental setup adopted consists of a custom-designed force plate platform for legged robots (see Supplementary Material). The Lilibot quadruped robot was monitored on the platform while moving forward using a trot gait. Data coming from the force plate platform were acquired at 20 Hz and synchronized with those ones acquired from the robot, in particular the joint motor currents, used as inputs for the network. The whole dataset acquired through a series of experimental trials on the robot is composed of 3,000 patterns properly divided between learning (80%) and test (20%). The idea to directly use the network previously trained in simulation with the newly acquired robot data was not pursued due to the differences in terms of input variables (i.e., motor currents instead of joint torques) and the actual set-up of the robot that has some differences if compared with the dynamic model from several aspects, for instance, the stepping frequency and the weight. Therefore, a new ESN was trained to design a soft sensor for GRF estimation. We considered a reduced network with 15 neurons in the reservoir to estimate the GRF associated to the front right leg, starting from the motor currents acquired from the Knee and Hip 2 joint motors of the same leg. The other hyperparameters adopted have remained unchanged from the Table 1. To filter out high-frequency noise in the motor currents, a 5th-order Butterworth filter with a cutoff frequency of 1.5 Hz was adopted. Figure 12 shows the normalized motor currents provided as input to the ESN, and the obtained GRF compared with the signals acquired from the force plate platform, applying a Z-score normalization. The GRF estimation for the first three steps is quite satisfactory, in fact, the testing performance obtained reports an MSE equal to 0.5 and a Pearson correlation coefficient (R) equal to 0.72. The behavior highlighted in the last step needs a brief explanation. The experiment here considered, as illustrated in the video of the robot walking on the sensorized platform included as Supplementary Material, reveals that the robot makes a slight turning to the right toward the end of the experimental trial. This change of direction affects the positioning of the legs as demonstrated in Figure 13 where a series of snapshots extracted from the robot experiment is reported. In the last picture of the sequence, the front right leg tip is placed on the boundary of two measurement units in which the force plate could not properly identify the GRF. The effect of this event is the missing of the GRF information as shown in Figure 12 after the time sample 80. In this case, the ESN network is still able to predict the GRF and the increment of the prediction error can be used as an indicator of a possible anomaly in either the sensing system or the robot behavior. This effect can be exploited thinking to a robot equipped with GRF sensors. The ESN model would represent an internal model capable of producing an efferent copy on which to evaluate the discrepancy between expectations and real conditions to identify anomalous situations.

Legged structures are good testbed to evaluate performance of neural models trying to estimate relevant information also acting as afference copy to be used to identify unforeseen situations like faults. A methodology for mapping local proprioceptive information (e.g., joint torque) into exteroceptive global information (e.g., GRFs) has been here presented. This methodology is based on reafference principle (Latash, 2021). The possibility to use recurrent neural networks (e.g., ESNs) to exploit their neurodynamics as well as embedded internal memory for robust state estimation (e.g., when missing input information) is another relevant aspect here addressed.

The obtained results demonstrate that the proposed approach is suitable to estimate the GRF in real quadruped robots walking on flat terrains. The differences between the real and simulated setups allow to conclude that the approach can be easily applied to different robot parametric configurations when input and output data can be acquired for the network training.

## 4. Conclusions

The methodology presented in this paper demonstrates the versatility of reservoir computing networks and exploits the ability of the reservoir to concurrently provide different analyses of the same input data and perform different static and dynamic mappings. This allows a dynamical layer constituting a high-dimensional sparse coding of the input features to be provided independently of the target output. The dynamical layer can be read out in many different ways concurrently. Moreover, the approach presented here is a clear example of a virtual sensor design. In fact, one of the functions of the ESN is to substitute actual force sensors with their estimated values using soft sensors. In addition, the structure does not use models that require analytical models of the robot, which can sometimes be complicated owing to the complexity of legged machines. In any case, the analytical representations can seldom take into account all the nonlinearities arising from the dynamic interplay between the different bodies in motion. The data-driven approach here is easy to implement and requires only data that can be acquired easily either in simulations or in simple experimental setups with the actual robot. Preliminary experiments carried out with the Lilibot quadruped demonstrate the effectiveness of the proposed approach in estimating the GRF of a leg starting from joint motor signals.

The possibility of adding multiple readout maps to extract the required information from the reservoir with simple and effective learning strategies demonstrated by the GRF and terrain classification is of great interest. It opens the way to the implementation of the proposed networks on dedicated hardware where high-level synthesis techniques, in conjunction with design automation allow the transformation of an offline-trained ESN algorithm into an embedded hardware accelerator. The next step in the further development of the proposed approach would be to evaluate the ESN for the estimation of the actual GRF signals and terrain classification recorded on the actual Lilibot robot in more complex scenarios.

## Data Availability Statement

The raw data supporting the conclusions of this article will be made available by the authors, without undue reservation.

## Author Contributions

MC developed the ESN, performed the simulations, and drafted the manuscript. LP led the development of the ESN, helped with data analysis, drafted the manuscript, and supervised the entire project. TS developed the robotic system, performed the robotic simulations, and recorded the data for the robot experiments. PA drafted the manuscript and supervised the entire project. PM cooperated with PA in the general direction of the project and reviewed the manuscript. All authors contributed to the conception and design of the work, approved the final version of the manuscript, and agreed to be accountable for all aspects of the work.

## Conflict of Interest

The authors declare that the research was conducted in the absence of any commercial or financial relationships that could be construed as a potential conflict of interest.

## Publisher's Note

All claims expressed in this article are solely those of the authors and do not necessarily represent those of their affiliated organizations, or those of the publisher, the editors and the reviewers. Any product that may be evaluated in this article, or claim that may be made by its manufacturer, is not guaranteed or endorsed by the publisher.

## Footnotes

^1^https://github.com/majianjia/nnom.
