# Supplementary material for: Echo State Networks for Estimating Exteroceptive Conditions From Proprioceptive States in Quadruped Robots
Source: Front Neurorobot. 2021 Aug 23;15:655330. doi: 10.3389/fnbot.2021.655330 (PMC8421012; doi:10.3389/fnbot.2021.655330)
Supplement: Supplementary file 3 [file Data_Sheet_1.PDF]

Supplementary Material

This supplementary document contains further details related to the dynamic simulations performed in the CoppeliaSim framework[1] of the Lilibot robot. Furthermore, a brief description of the experimental platform adopted to acquire data from the real robot is reported. Videos showing the simulated and real robot in action are also provided.

1 SIMULATION RESULTS

The dynamical model of the Lilibot robot was simulated in the CoppeliaSim framework to acquire the input (i.e., joint torque signals) and targets (i.e., ground reaction forces) used in the proposed ESN. Figure S1 depicts the terrain configuration adopted to acquire the sensory information of the simulated Lilibot.

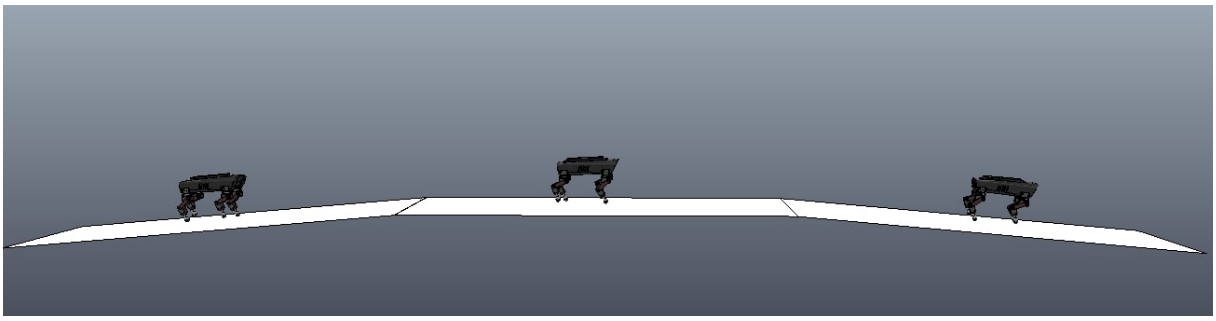

Figure S1. Dynamic simulation scenario used to test the Lilibot on terrains with different slopes: downhill ($-5°$ slope), flat ground ($0°$ slope), and uphill ($5°$ slope).

The results included in the manuscript for the GRF estimation refer to an ESN training phase performed using the data acquired on flat terrain and a testing phase, including downhills and uphills with slopes of $\pm 5°$. To further investigate the generalization capabilities of the proposed network, a new testing dataset has been considered analyzing the robot behaviour on steeper slopes. Figure S2 shows the simulation scenarios used to test the ESN on terrains with slopes of $\pm 15°$.

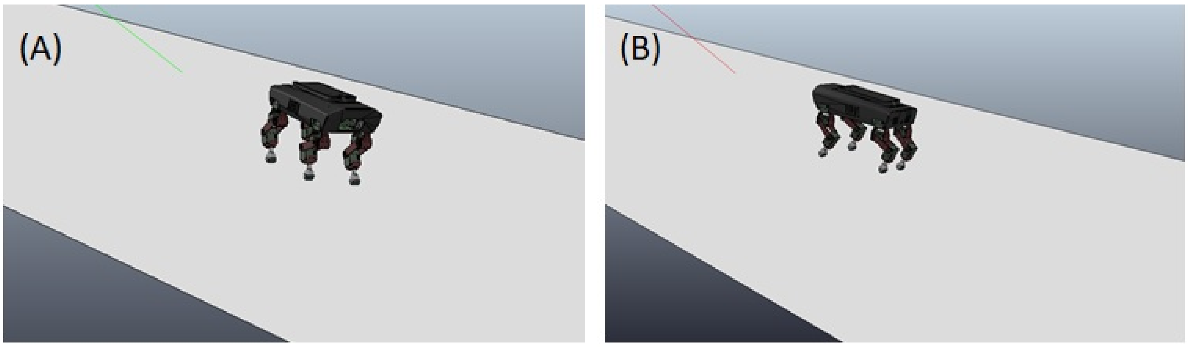

Figure S2. Lilibot walking on downhill ($-15°$ slope) and uphill ($15°$ slope). Videos related to the corresponding simulations are included in the Supplementary material.

[1] https://www.coppeliarobotics.com/

	Test on $\pm 10^\circ$ slope				Test on $\pm 15^\circ$ slope			
	**FL**	**FR**	**HL**	**HR**	**FL**	**FR**	**HL**	**HR**
**MAE**	0.035	0.039	0.056	0.055	0.037	0.036	0.070	0.058
**MSE**	0.0041	0.0045	0.0051	0.0044	0.0044	0.0038	0.0076	0.0049
**R**	0.97	0.97	0.95	0.96	0.97	0.97	0.90	0.94

Table S1. Analysis of the estimation error of the ESN learned on flat terrains and tested on uphill and downhill terrains with $\pm 10^\circ$ and $\pm 15^\circ$slope. The four legs are labelled as: front left (FL); front right (FR); hind left (HL); hind right (HR).

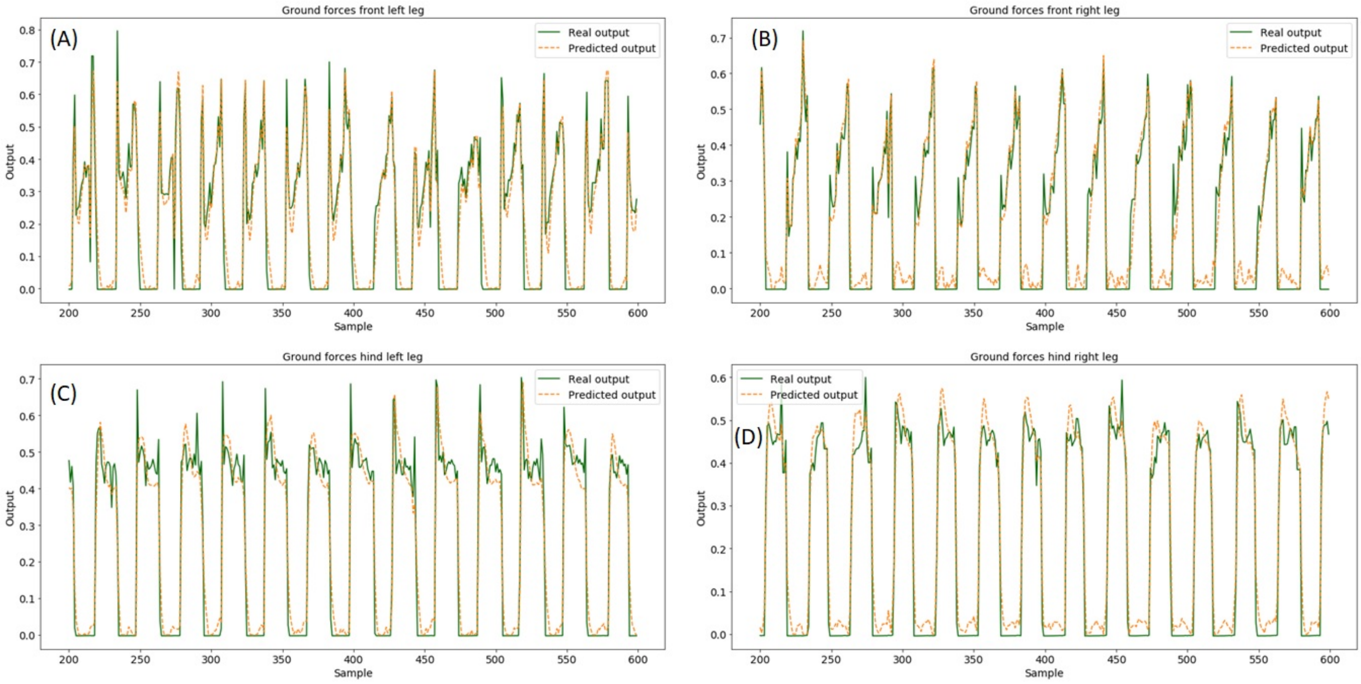

Figure S3. Comparison between estimate and actual GRF during testing on uphill with a 10° slope).

The obtained results are summarized in Table S1 where the mean absolute error (MAE), the mean squared error (MSE) and the Pearson correlation coefficient (R) are reported for each leg. The time evolution of the GRF comparing the estimated value and the actual one is reported in Figure S3 and S4 where the results obtained on uphill terrain with a 10° and 15° slope are shown respectively.

Even if the network was trained using only data from flat terrains, it was able to generalize the results also for different terrain configurations.

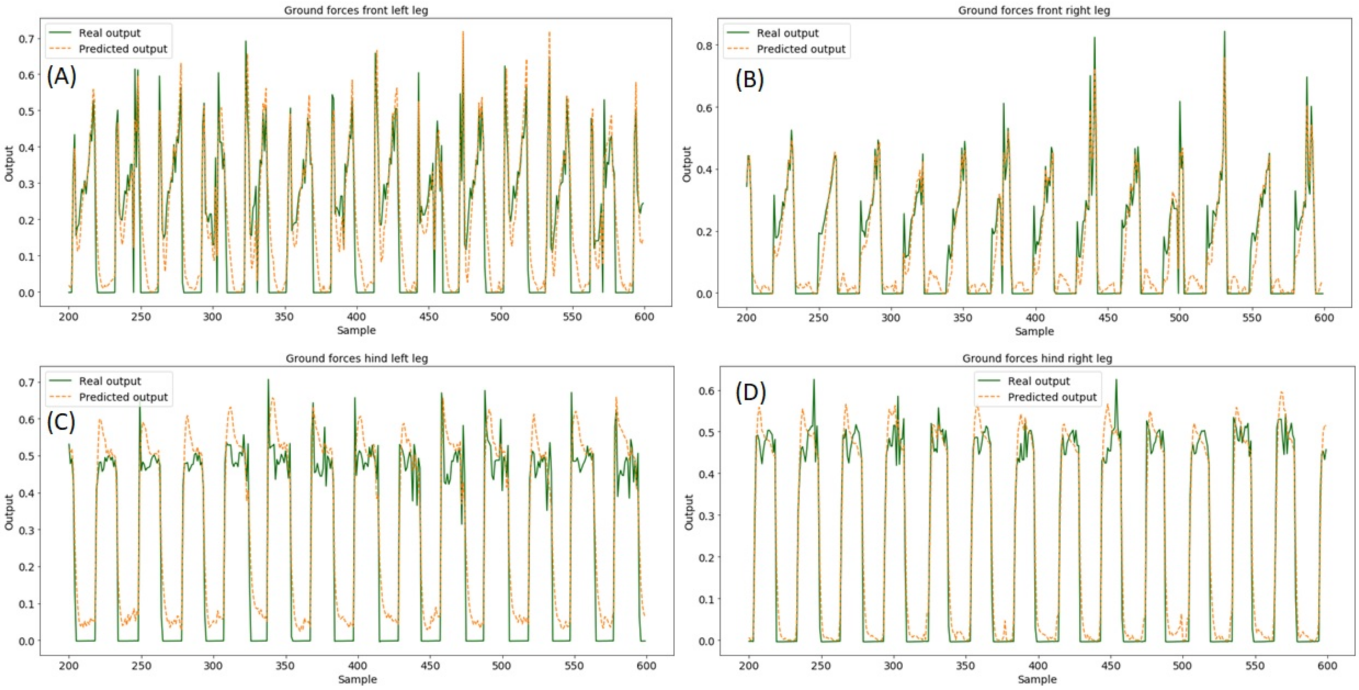

Figure S4. Comparison between estimate and actual GRF during testing on uphill with a 15° slope).

2 ROBOT EXPERIMENTS

The experimental setup adopted to test the ESN for GRF estimation on the quadruped robot Lilibot consists of a custom-designed force plate platform as shown in Figure S5.

The acquired signals used as input for the ESN are the motor currents. The trend of these acquired signals for the front right leg is reported in Figure S6. A Min-max normalization in the range [-1 1] was applied to the motor current signals. A low pass filter was applied to filter out the high frequency noise present in the motor signals before providing the data to the ESN.

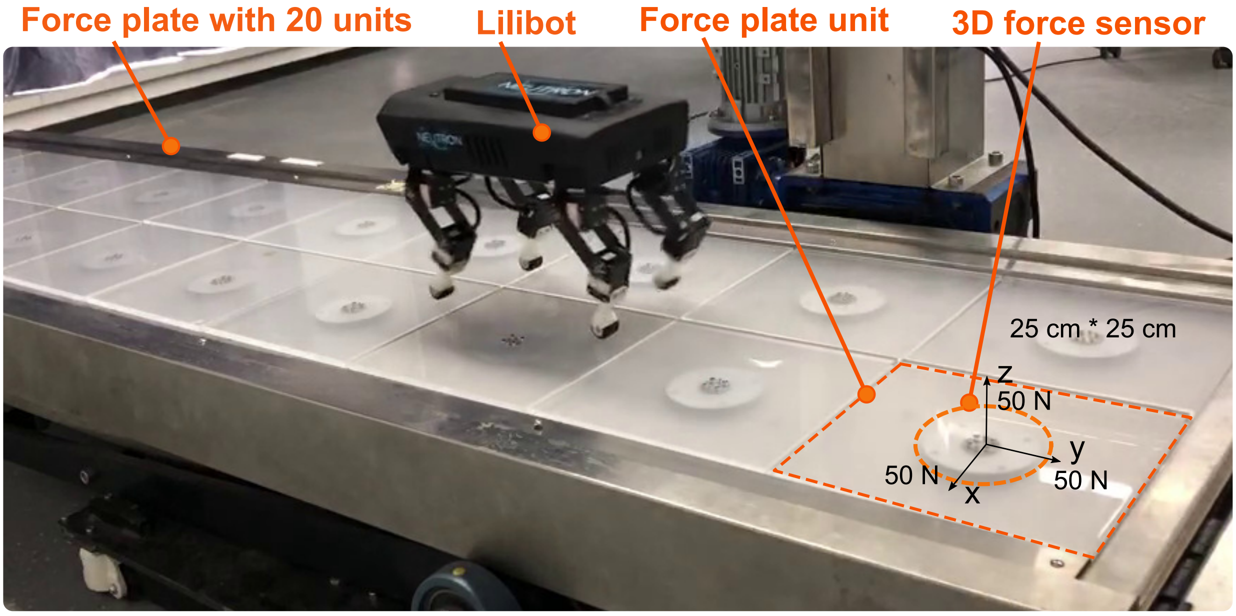

Figure S5. Experimental setup of the real robot walking on a force plate for measuring the GRFs. The GRFs were measured through a custom-designed force plate platform with 20 measurement units when the robot walked on the plate. Each measurement unit has a 25×25 cm^2 bearing area and a 3D force sensor with a range of -50 to 50 newton along every axis. The update frequency of the force data can be up to 150 Hz.

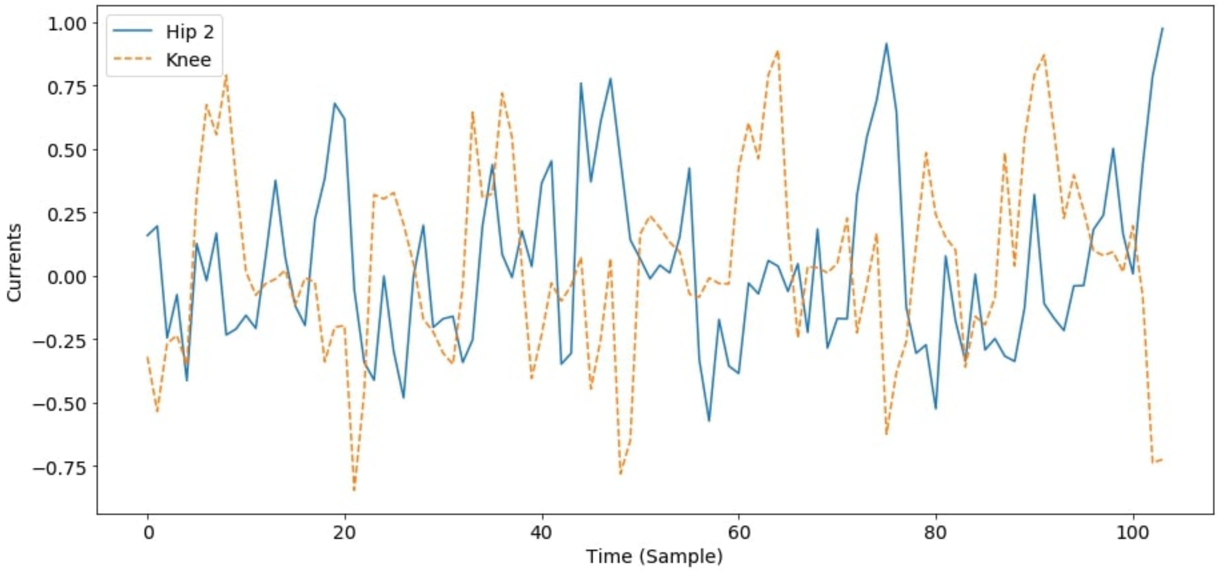

Figure S6. Normalized motor currents on the Hip 2 and Knee robot joints before the application of the low-pass filter as reported in the manuscript. The sampling rate is 20 Hz.
